# A five-year retrospective study of the epidemiological characteristics and visual outcomes of patients hospitalized for ocular trauma in a Mediterranean area

**DOI:** 10.1186/1471-2415-8-6

**Published:** 2008-04-22

**Authors:** Salvatore Cillino, Alessandra Casuccio, Francesco Di Pace, Francesco Pillitteri, Giovanni Cillino

**Affiliations:** 1 Department of Clinical Neuroscience, Ophthalmology Section, Palermo University, Italy

## Abstract

**Background:**

To determine the epidemiological characteristics and visual outcome of ocular trauma in southern Italy.

**Methods:**

All cases of ocular trauma admitted to Department of Ophthalmology of Palermo University, Italy, from January 2001–December 2005 were retrospectively reviewed for open- or closed-globe injury (OGI or CGI). Data extracted included age, sex, residence, initial and final visual acuity (VA), cause and treatment of injury, hospitalization. The injuries were classified by Ocular Trauma Classification System (OTCS) and Birmingham Eye Trauma Terminology (BETT). We also referred to the Ocular Trauma Score (OTS) in evaluating the final visual outcome.

**Results:**

Of the 298 eyes, there were 146 OGI and 152 CGI. Fifty eyes (16.8%) had an intraocular foreign body (IOFB). The annual incidence of eye injuries was 4.9 per 100,000. Most injuries occurred in men (84.6%, p < 0.0005), with an average age of 33.0 vs. 49.9 for women (p = 0.005). Cause of injury differed significantly by gender (p = 0.001) and urban vs. rural location (p = 0.009). The most frequent causes in men were outdoor activities related injuries (30.9%), work-related (25.4%), and sport-related (17.5%), and in women were home-related (52.2%) and outdoor activities related injuries (30.4%). In urban areas, road accidents were more frequent; in rural areas, work-related injuries were more frequent with a greater rate of IOFBs than in urban areas (p = 0.002).

The incidence of OGI and CGI differed in work-related injuries (p < 0.0005), sport-related injuries (p < 0.0005), and assaults (p = 0.033). The final visual acuity was 20/40 (6/12) or better in 144 eyes (48.3%), 20/40–20/200 (6/12–6/60) in 90 eyes (30.2%), and <20/200 (6/60) or less in 46 eyes (15.5%). Eighteen eyes (6%) had a final acuity of no light perception. Of those eyes that presented with hand motion vision or better, 220 (86.6%) had a final vision of better than 20/200 (6/60). Initial visual acuity was found to be correlated with final visual acuity (Spearman's correlation coefficient = 0.658; p < 0.001). The likelihood of the final visual acuities in the OTS categories was correlated to that of the OTS study group in 12 of 14 cases (85.7%).

**Conclusion:**

This analysis provides insight into the epidemiology of patients hospitalized for ocular trauma. The findings indicate that ocular trauma is a significant cause of visual loss in this population.

## Background

Open globe ocular injuries constitute a major cause of visual morbidity worldwide [1], with significant socioeconomic impact [1,2]. Ocular trauma is an important, preventable, worldwide public health problem [3]. Every year, approximately 2 million eye injuries occur in the United States, of which, more than 40 thousand result in permanent visual impairment [4,5]. Prior studies in which the incidence of eye injury has been examined have produced varied results, in part because of study design differences [4,6-11]. When considering eye injuries requiring hospital admission, rates have ranged from 8 to 57 per 100,000 [4,6-11]. Despite the heterogeneity of results, these studies provide important informations regarding the burden of eye injury. However, they have all been limited to a single year or narrow time frame making it difficult to determine trends in injury rates over time. A population-based study reported in U.S.A. a prevalence rate of 19.8% and an average annual incidence rate of 3.1 × 1000 population [12]. In a more recent study from 1992 through 2002, the incidence of eye injury declined overall and the estimated rate of eye injury ranged from 8.2 to 13.0 per 1000 population [13]. Other studies performed in Australia have estimated the annual incidence of all injuries at 15.2 per 100,000 in urban settings [14], and 11.8 per 100,000 in rural settings [15].

While the incidence of ocular trauma has been described in the United States [4,6,9,10,12,13], the United Kingdom [8,16], Sweden [17], and Greece [18], it has not been well studied in other industrialized countries, like Italy, where clinical research on ocular trauma is limited to the pediatric populations and sportsmen [19-21] and no studies are available on adults hospitalized with ocular trauma. From a public health and injury prevention perspective, current information on eye injuries rates is needed to develop effective plans for disseminating eye injury prevention materials to the public and to earmark adequate funding for these initiatives.

This study retrospectively analyzes the epidemiology of patients with ocular trauma presenting to the Department of Ophthalmology of Palermo University, Italy, and evaluates their visual outcomes. We emphasize that Palermo is the regional capital of Sicily, which is the largest Mediterranean island characterized by a strategic central geographical position. Hence, these data may be representative for any surrounding Mediterranean area.

## Methods

The area of investigation includes the Western Sicily's health district. The city of Palermo, capital of the province and of the Sicily region, is located on the north coast of the western Sicily (geographic coordinates 38°08 north and 13°23 east) and covers about almost 180 square km (represented by a built-up center of about almost 39 square km), at a mean altitude of about 14 meters above sea level. The current population is just over 730,000. The economy includes agriculture (citrus fruits, vegetables), industries, handicraft, commerce and trade, tourism, and services. The province of Palermo covers 5,016 square km with about 1,250,000 inhabitants, while the Sicily region covers 25,700 square km with about 5,200,000 inhabitants.

The study included all patients with ocular and orbital trauma at the Department of Ophthalmology of Palermo University, Italy, over a 5 year period from January 2001 through December 2005. This ophthalmic unit is the major adult eye trauma centre which serves as a major referral center for a large geographic area (12,600 square kilometers) of the Western Sicily's health district, with a population of approximately 1,950,000 inhabitants. During the study period (2001 to 2005), the population was stable and there were not significant changes in sex and age structure of the population.

As a centre of excellence in eye care, the Department of Ophthalmology of Palermo University offers both emergency eye care and specialized care for patients of all ages with specific and complicated ocular or orbital diseases and conditions with a 24-hour Ophthalmic Emergency Department. In Palermo there is another public hospital as center for major trauma but not considered referral center and without a 24-hour Ophthalmic Emergency Department. This offers the opportunity to analyze ocular traumatic injuries in an well-defined study area. The study was approved by the Institutional Ethics Committee of Faculty of Medicine, Palermo University.

In this study, ocular injury was defined as any injury affecting the eye or adnexa requiring hospital admission and having a principal discharge diagnosis from the International Classification of Diseases, Tenth Revision, Clinical Modification (ICD-10-AM). Patient records were identified by computer search using codes from the ICD-10-AM and the diagnostic codes were chosen to be identical to those used by Tielsch et al. [6] and Klopfer et al. [9].

Records from 290 patients were reviewed for open or closed globe injury, and classified by the Standardized international classification of ocular trauma (Birmingham Eye Trauma Terminology, BETT) [22,23] as those involving blunt force, resulting in contusion (closed globe injury) or rupture (open globe injury), and those involving sharp forces, resulting in lamellar laceration (closed globe injury) or penetrating, perforating, and intraocular foreign body laceration (open globe injury). Moreover, the Ocular Trauma Classification System (OTCS) [24] classified the ocular trauma on the basis of visual acuity, anatomical location of wound, mechanism of injury, and presence of an afferent pupillary defect.

Records of the initial visit were assessed for visual acuity, mechanism of injury and the zone of injury. Zone I, II, and III were, respectively, from the anterior to the posterior pole of the globe [24].

We also referred to the Ocular Trauma Score (OTS) [25] in evaluating the final visual outcome. This index allows prediction of the visual outcome in a population with ocular trauma according to the initial visual acuity, type of injury, and associated findings. Certain numerical values rendered to the OTS variables (visual acuity, rupture, endophthalmitis, perforating injury, retinal detachment, and afferent pupillary defect) at presentation were summated and converted into OTS categories; the likelihood of the final visual acuities (NLP, LP/HM, 1/200 to 19/200, 20/200 to 20/50, and ≥ 20/40) in the OTS categories (1 through 5) in this study group were calculated and compared with those in the OTS study group.

Patient's data extracted included age, sex, place of residence, date and cause of injury, initial and final best-corrected (Snellen) visual acuity, anatomical site, location and nature of injury, primary and secondary repair, adjuvant treatment, duration of hospitalization and follow-up. On the basis of location of eye injury we have classified the data in six groups: work related injuries, home related injuries (by falls or by cut/piercing objects, struck against or by, etc.), recreational/sport related injuries, road accident related injuries, assaults related injuries, and other various outdoor activities related injuries (included other accidental trauma occurred in outdoor environment). The initial visual acuity was the acuity measured on presentation to the hospital; the final visual acuity was taken on the most recent outpatient visit.

### Statistical analysis

Data were analyzed with Epi Info version 6.0 (CDC, Atlanta, GA, US) and SPSS version 14.0 (SPSS, Inc., Chicago, IL, US). For each year (2001–2005), eye injury rates were calculated, using denominators obtained from the Italian National Institute of Statistics (ISTAT). As everyone in the population was theoretically at risk for eye injury, this denominator is appropriate. Frequency distributions were created for injury type and cause.

Statistical analysis of quantitative data, including descriptive statistics, parametric and non-parametric comparisons, was performed for all variables. Frequency analysis was performed by the chi-square test. One-way analysis of variance (ANOVA) was used to evaluate differences in parametric variables. Correlation analysis for initial and final visual acuity was performed with Spearman's test. Categorical evaluations were done for the numeric scores representing the likelihood of the final visual acuity in the OTS study and this study group. Chi square test or Fischer exact test were used as appropriate. All P-values were two-sided and P-values less than 0.05 were considered statistically significant.

## Results

There were 298 eye injuries from 290 patients over the 5-year period (44 cases in 2001; 72 cases in 2002; 56 cases in 2003; 66 cases in 2004 and 60 in 2005 year) (Table 1). There was no significant change in annual rates of injuries during the five year period. The minimum follow up was 4 months. There was a similar incidence of open globe injuries (146 eyes) and closed globe injuries (152 eyes). Fifty eyes (16.8%) had an intraocular foreign body (IOFB). There was no significant difference in frequency of right vs. left eye injuries (160 right vs. 138 left). There were eight bilateral penetrating injuries.

**Table 1:** Characteristics of patients hospitalized with ocular trauma diagnosis over a 5-year period (January 2001–December 2005)

Crude annual hospitalized injuries incidence (per 100,000)	4.9
Urban/rural incidence (per 100,000)	4.2/5.5
Left/right eye	138/160
Open/closed globe	146/152
Male:female ratio	5.5/1
Age (years, mean ± SD)	
Total	35.6 ± 21.0
Men	33.0 ± 19.6
Women	49.9 ± 23.1
Mean duration of hospitalization (days)	4.2 (range: 1–14)
Diagnosis	ICD-10-AM Code
Orbital floor fractures (including blowout fractures)	6
Open wounds of ocular adnexa	2
Open wounds of globe	118
Superficial wound of eye and adnexa	32
Contusion of eye and adnexa	122
Foreign body on external eye	6
Burn confined to eye and adnexa	4
Injury to optic nerve and pathways	4
Injury to oculomotor, trochlear, and abducens nerve	4

ICD-10-AM = International Classification of Diseases, Tenth Revision.

Based on ISTAT population census data, the average annual rate of eye injuries for our health district was 4.9 per 100,000 (95% CI, 4.8–5.0) – 2.4 per 100,000 (95% CI, 2.35–2.45) for open globe injuries and 2.5 per 100,000 (95% CI, 2.4–2.55) for closed globe injuries. The rates in urban (4.2/100,000; 95% CI, 4.1–4.3) and rural areas (5.5/100,000; 95% CI, 5.4–5.65) were not statistically different.

**Figure 1:**
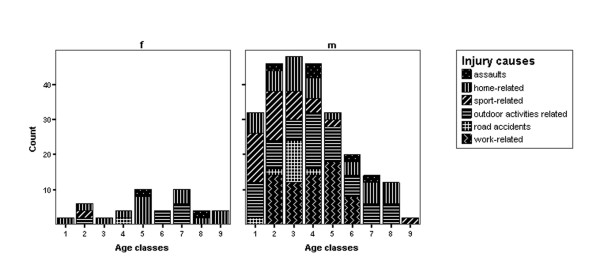
Frequency of causes of eye injury by age and sex.

The majority of all injuries occurred in men (84.6%; male:female ratio = 5.5:1, p < 0.0005; Pearson's chi square test). The average age was 35.6 ± 21.0 years. There was a significant difference between the average age for men (33.0 ± 19.6 years) and for women (49.9 ± 23.1 years, p = 0.005; ANOVA test). The majority of injuries occurred in males less than 50 years (68%), with a slight predominance in the second to fifth decade. Thirty-two patients (11%) were male children under 10 years old (Figure 1).

There was a correlation between location of injury and gender (p = 0.001; Pearson's chi square test) (Figure 1). The outdoor activities related injuries accounted for 30.9% of injuries in men, followed by work related injuries (25.4%) and sport related injuries (17.5%). The most frequent cause of ocular trauma in women was home related work (52.2%), followed by the outdoor activities related injuries (30.4%). Assaults accounted for 4.5% of all injuries, and alcohol use was documented in 71.4% of these.

**Figure 2:**
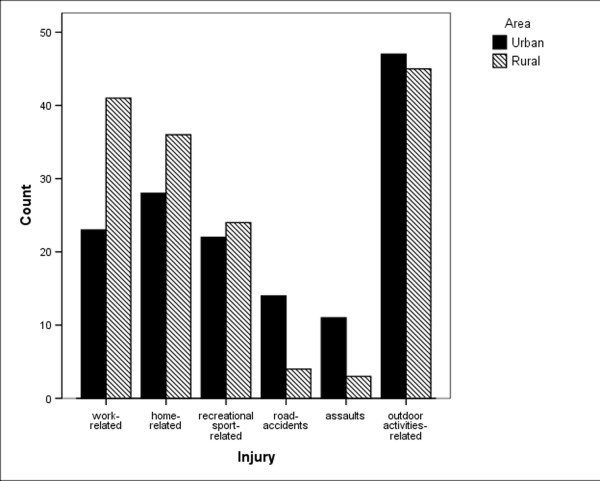
Frequency of causes of eye injury by urban/rural area.

**Figure 3:**
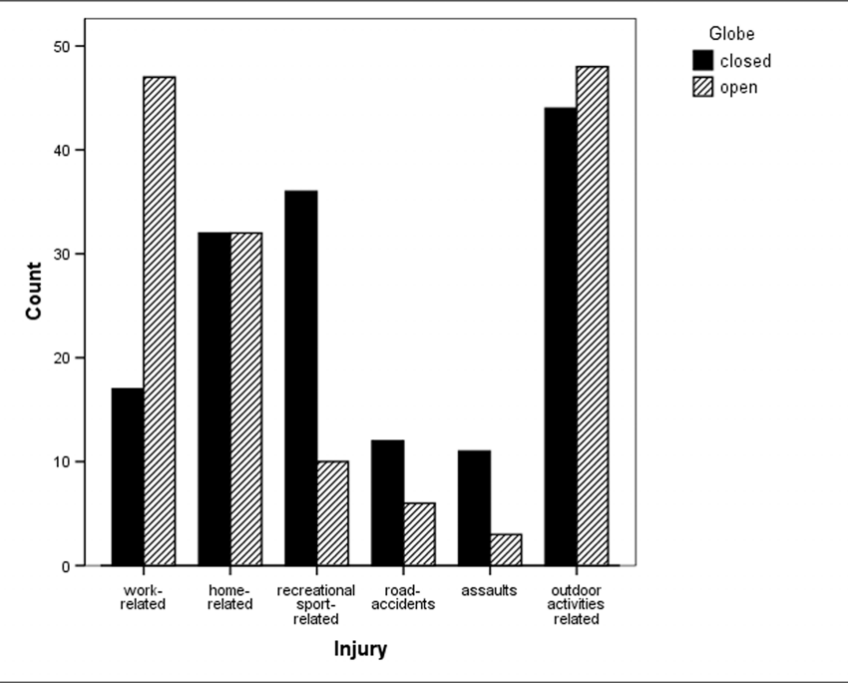
Frequency of causes of eye injury by open/closed globe.

The causes of eye injuries by urban/rural area, and by open/closed globe are shown in Figures 2 and 3. The causes of the injury were significantly associated with geographic location of the residence of patients (p = 0.009; Pearson's chi square test). In urban areas, road accidents and assaults were more frequent, (78% vs. 22% for road accidents, 71% vs. 29% for assaults, in urban vs. rural, respectively). In rural areas, work related injuries were more frequent (66% vs. 34% in rural vs. urban, respectively). In the rural area a greater number of IOFBs were found (p = 0.002; Pearson's chi square test).

There was a significant difference in the frequency of open and closed globe injuries in work related injuries (73% open vs. 27% closed, p < 0.0005; Pearson's chi square test), sport related injuries (22% open vs. 78% closed, p < 0.0005; Pearson's chi square test), and in assaults (21% open vs. 79% closed, p = 0.033; Pearson's chi square test). Eye protection was present in 25% and 71% and was absent in 22% and 9% in open and closed globe injuries, respectively. Such data were not available in 53.0% of open and in 20.0% of closed globe injuries. In open globe injuries, the anatomical site of the entrance wound was corneal in 40%, scleral in 24%, corneoscleral in 22%, and limbal in 14%.

The clinical and surgical procedures performed are summarized in Table 2. The most common primary surgery concerned reconstitution of the globe integrity with reposition or excision of ocular contents. All surgeries occurred within 5 hours of presentation to the hospital. The majority of primary repairs of open globe injuries involved suture of the wound with or without excision of prolapsed intraocular contents or lensectomy (58.9%). Four primary enucleations were performed for severely ruptured globes. All these injuries were due to assault. Six open globe injuries, four of which were from assault, required secondary enucleations. Among the open globe injuries, lensectomy was performed in nine cases (6.3%) during primary repair and in 14 cases (9.5%) as a secondary procedure. Forty-nine of the 146 open globe injuries required further surgery, 26 cases by vitreoretinal procedures.

**Table 2:** Procedure employed for treatment of hospitalized patients with ocular trauma

Procedure	Frequency
Medical therapy and simple repair of ocular injuries (including primary repair of conjunctival, corneal, and scleral wounds)	140
Complex repair of ocular injuries (including repair of large corneo-scleral wounds, excision of prolapsed uveal tissues, removal of lens, and anterior vitrectomy)	108
Removal of IOFB from the anterior chamber (including other allied anterior segment procedures)	24
Removal of IOFB from the posterior chamber (including complex posterior segment surgeries, such as a concomitant pars plana vitrectomy with or without lensectomy)	26

IOFB = Intraocular foreign body.

Contusions involving the anterior segment were the most common closed globe injuries and had the best prognosis. Treatment of hyphemas ranged from bed rest to antifibrinolytic agents.

Adjuvant treatments included intravenous antibiotics (cephalothin and gentamicin, given to 86% of patients), topical antibiotics and steroids (given to 90%). Only two cases (both open globe injuries) received intravitreal antibiotics.

Two of the patients with open globe injuries developed endophthalmitis and the infection's signs were recognized at patient presentation. In both cases there was a strong association between endophthalmitis and the mechanism of injury, which included an accidental lesion with a piece of wood and a punch. These cases had vitrectomy and received intravitreal antibiotics at the primary repair. The culture results reported Staphylococcus spp. isolates in both patients. Although treated aggressively, visual outcome was poor in both cases, which achieved hand motion after long term follow up.

The final visual acuity was 20/40 (6/12) or better in 144 eyes (48.3%), 20/40–20/200 (6/12–6/60) in 90 eyes (30.2%), and <20/200 (6/60) or less in 46 eyes (15.5%). Eighteen eyes (6%) had a final acuity of no light perception. Of those eyes that presented with hand motion vision or better, 220 (86.6%) had a final vision better than 20/200 (6/60). Those patients with an initial acuity of no light perception had a poor prognosis. Initial visual acuity was found to be correlated with final visual acuity (Spearman's correlation coefficient = 0.658; p < 0.001).

Except for the statistically higher and lower ratio in the final visual acuity category in the OTS score 1 and 2 respectively, our results were similar to those in the OTS study (Table 3). The likelihood of the final visual acuities (NLP, LP/HM, 1/200 to 19/200, 20/200 to 20/50, and ≥ 20/40) in the OTS categories of this study group was correlated to that of the OTS study group in 12 of 14 cases (85.7%) (Table 3).

**Table 3:** Likelihood of the final visual acuity category by the OTS score in comparison with the Ocular Trauma Score (OTS) Study (data OTS study/study group)

**Raw score sum**	**OTS score**	**NLP**	**LP/HM**	**1/200–19/200**	**20/200–20/50**	**≥ 20/40**
**0–44**	**1**	73	17	7	2/100 *	1
**45–65**	**2**	28/23	26/27	18/2 *	13/26	15/22
**66–80**	**3**	2	11/16	15/8	28/45	44/31
**81–91**	**4**	1	2	2	21/35	74/65
**92–100**	**5**	0	1	2	5/3	92/97

* p < 0.05

There was a significant difference in final visual acuity between open and closed globe injuries (p = 0.036; ANOVA test). In the majority of open globe injuries (mostly Zone III), there was a poor visual prognosis, with final visual acuity of 20/200 (6/60) or worse. Among Zone I and II injuries, 32.6% had a final visual acuity of 20/40 (6/12) or less, and 21.9% had a final visual acuity of 20/200 (6/60) or worse. Among closed globe injuries, all Zone I injuries had a final visual acuity of 20/40 (6/12) or better (63%), while of the Zone II and Zone III injuries, 56.4% had a final visual acuity of 20/40 (6/12) or better and 12.3% with a final visual acuity of 20/200 (6/60) or worse.

Of the 50 eyes with an IOFB, 32 (64%) had a final visual acuity of 20/40 (6/12) or better. In 22 eyes (44%), the initial visual acuity was less than 20/200 (6/60).

Average duration of hospitalization was 4.2 days (range: 1–14 days) – 4.6 days (range: 1–14 days) for open globe injuries and 3.4 days (range: 1–9 days) for closed globe injuries (p = 0.002; ANOVA test). There was no significant variation in the day of the week, month, or season in which injuries occurred.

## Discussion

Despite the fact that ocular trauma is an important cause of monocular blindness worldwide, scant information is available regarding its epidemiology outside the United States and a few other developed countries [3,26].

Estimates of the rate of ocular trauma are highly dependent on its definition and the source of data [6,9,10,26,27]. However, from a public health perspective, sight threatening injuries are those of greatest concern. Hospital discharge data provide a useful source of such information.

To our knowledge, this is the first study examining the epidemiology of hospitalized ocular trauma in a population in Italy. Our study provides insights on the epidemiology of ocular trauma in hospitalized patients in Italy and supports the findings that ocular trauma may represent a significant cause of visual loss in this population [3,26].

The average annual rate of eye injuries found in the Western Sicily health district, is similar to rates reported in other industrialized countries [28,29]. Our rates of eye injuries are less than those reported by Tielsch et al. [6] in Maryland in 1989 (13.2 per 100,000), Klopfer et al. [9] in the United States in 1992 (13.2 per 100,000), and Chen et al. in Michigan in 2006 (12.0 per 100,000) [27]. This difference may be related to the higher threshold for hospitalization in closed globe injury. Nonetheless, the admission rate and surgical rate for open globe trauma remained stable over the five years of this study. Also, the Department of Ophthalmology of Palermo University, Italy, is a major eye trauma centre in Western Sicily and the majority of cases seen are severe. However, our rates are similar to rates reported from McGwin et al. in 2006 for inpatient group [13].

Reported incidence and prevalence ratios between men and women range from two to over five [6,9,10]. In our study, men were affected more than women, with the outdoor environment, workplace and the home the most commonly reported locations of trauma [1,7,14,30,31]. The majority of injuries occurred in males, less than 50 years of age (68%), with a slight predominance in the second to fifth decade. A male preponderance universally reported and thought to be related to occupational exposure, participation in dangerous sports and hobbies, alcohol use and risk-taking behavior [7,14,29-34]. The higher risks in men up to 70 years reflect a combination of a high incidence of work related, assault related, sport related, and motor vehicle crash related ocular injuries. The similar risks observed between the sexes at the older age range appears to be related to changes in lifestyle and occupational patterns by men after age 70 years [6,9,10].

Lack of eye protection was a risk factor identified in previous studies [34], with at least 22% of patients with open globe injuries and 9% with closed globe injuries failing to wear eye protection. It is likely that most of the high velocity fragment injuries could have been prevented by the use of polycarbonate protective eyewear [34].

Children and students (first and second decade) represented 28.2% of the injuries, reinforcing the need for prevention of childhood injuries within the home and during sports [35].

Eighty-four ocular injuries (28.2%) were associated with injuries of the ocular adnexa, orbital wall fractures, or other non ocular structures, demonstrating that ocular trauma requires a multidisciplinary approach.

In our study, open globe injuries had poorer visual prognosis than closed globe injuries. In a multivariate analysis of prognostic factors in penetrating eye injury, Sternberg et al. noted that a good initial vision statistically correlated with a good final vision [36]. This is consistent with other studies, and is the most important prognostic factor when counselling patients after injury [36-41]. The most common open globe injuries were anterior penetrating injuries, which have been associated with a good visual prognosis [36-38]; open injuries involving the posterior globe had a poor prognosis. Except for the statistically higher and lower ratio in the final visual acuity category in the OTS score 1 and 2 respectively, our results were similar to those in the OTS study (85.7%). Because this study is a retrospective and nonrandomized one, possible treatment selection bias may have affected our results.

Fifty eyes (16.8%, 34.2% of open globe injuries) had an IOFB. Complications included two cases of endophthalmitis, both in elderly patients. The literature indicates that post traumatic endophthalmitis is not common, complicating approximately 5% of cases [42,43], which is consistent with our study.

Different from other studies [14,41], we found that open globe injuries had a similar frequency to closed globe injuries. This may reflect the status of the Department of Ophthalmology of Palermo Hospital University as a center for severe eye trauma. Moreover, in recent years closed globe trauma has been treated in an ambulatory setting. Contusions involving the anterior segment were the most common closed globe injuries and had the best prognosis. The incidence of secondary bleeds was low (<1%), consistent with past studies [39]. Wounds confined to the cornea had the best visual outcome in our study, as has been noted in previous studies [36]. This probably reflects both the mild severity of the initial injury and the ability of the anterior segment to heal.

A large proportion of the patients were from rural areas (51.7%), also reported by others [7,44], and this reflects a greater exposure to dangerous occupations in rural areas. As opposed, in urban areas, there were more injuries related to motor vehicle crashes and assaults.

A limitation of a retrospective study of this type is that the data are static, while the epidemiology of ocular trauma is dynamic. The data were derived from a single hospital's records, the number of people at risk could not be accurately determined and a bias of underestimating the true prevalence of ocular trauma may have occurred due to the loss of many of the cases of minor trauma or those found in polytraumatized patients by motor vehicle trauma. However, these limitations do not significantly affect the major findings of this study.

Aside from visual impairment, eye injury is known to cause significant morbidity in terms of pain, psychosocial stress, and economic burden. The cost of work-related eye injury is estimated at 1 to 3 billion dollars annually. [13] With proper information and education, up to 90% of eye injuries and a significant amount of its burden are preventable [45]. The results of our study indicate relatively low rates of eye injury. However, certain segments of the population continue to be at high risk (e.g., males and persons aged 50 and younger) and are those to whom prevention resources should be directed.

## Conclusion

The results of this study suggest the need to explore strategies to minimize ocular trauma as a priority. Eye care programs targeting high-risk ocular trauma groups may need to consider ocular trauma as a priority in eye health awareness strategies to reduce blindness due to trauma. Further, trauma is usually not a random event and the groups in which trauma occurs need to be targeted with preventive strategies. The importance of eye protection, which is probably not fully appreciated by the exposed population in our area, should be emphasized to those at high risk. Furthermore, such use should be recorded in the medical record. Based upon our findings, health education and safety strategies, which have traditionally targeted the workplace, sports, and other high-risk activities, should also target high-risk activities at home.

A standardized reporting system, United States Eye Injury Registry (USEIR) surveillance, as exists in other countries [46], would help to evaluate changes in the epidemiology of eye trauma over time and provide population-based longitudinal data for preventive strategies. Prospective studies to better identify prevention strategies have previously been suggested [30] and should be encouraged.

## Competing interests

The authors declare that they have no competing interests.

## Authors' contributions

SC conceived of the study and participated in its design and coordination. AC participated in the design of the study and performed the statistical analysis. FDP participated in the design and drafted the manuscript. FP participated in the design of the study and helped to draft the manuscript. GC participated in the design of the study and helped to draft the manuscript. All authors read and approved the final manuscript. No funding was obtained by any of the authors for this study.

## Pre-publication history

The pre-publication history for this paper can be accessed here:


